# Recent advances in cancer outcomes in HIV-positive smokers

**DOI:** 10.12688/f1000research.12068.1

**Published:** 2018-06-11

**Authors:** Sabina Ranjit, Santosh Kumar

**Affiliations:** 1 Department of Pharmaceutical Sciences, College of Pharmacy, University of Tennessee Health Science Center, 881 Madison Avenue, Room 456, Memphis, TN, 38163, USA

**Keywords:** HIV, tobacco smoking, cancer

## Abstract

HIV-infected smokers are at relatively higher risk of cancer than HIV-infected non-smokers. HIV weakens the immune system and renders infected individuals more vulnerable to the carcinogenic effects of smoking. HIV-infected smokers suffer more aggressive forms of cancers than do non-smokers because of the cumulative effects of the virus and cigarette smoke carcinogens. The major types of cancer observed in HIV-infected smokers are lung, head and neck, esophageal, anal, and cervical cancers. In this review, we will discuss the recent advances in cancer outcomes, primarily in terms of cancer incidence, prevalence, and progression in HIV patients who are smokers.

## Introduction

About 1.2 million people are currently suffering from HIV/AIDS in the US. The Joint United Nations Programme on HIV/AIDS (UNAIDS) reveals that deaths resulting from HIV infection dropped by 45% between 2005 and 2015 (UNAIDS 2015 report). The decrease in HIV-related deaths can be attributed to the advancements in combination antiretroviral therapy (cART). cART has elongated the life spans of people living with HIV/AIDS (PLWHAs). However, the burden of cancer (especially non-Hodgkin’s lymphoma, Kaposi’s sarcoma, anal cancer, and lung cancer) has substantially increased in those patients (by about 50%) ^1^. Cancer-related deaths in PLWHAs have increased by 8% within the span of a decade from 2000 to 2010 ^2^.

HIV weakens the immune system of infected patients and makes them vulnerable to cancer-associated viruses such as human herpesvirus 8 (HHV8), Epstein–Barr virus (EBV), and human papillomavirus (HPV) ^3, 4^. These viruses cause Kaposi’s sarcoma, non-Hodgkin’s lymphoma, and cervical cancer, respectively. As the prevalences of these cancers are specifically higher in patients with HIV, they are referred to as AIDS-defining cancers (ADCs) ^5^. Patients with HIV are also susceptible to other non-ADCs (NADCs) such as Hodgkin’s lymphoma and anal, skin, liver, kidney, lung, mouth, throat, and testicular cancers ^6^. According to the recent study by the Division of Cancer Epidemiology and Genetics, National Cancer Institute, USA (2014), the number of ADC cases was observed to have decreased over the period from 1996 to 2010 while the number of NADCs such as anal, liver, and prostate cancer was found to have increased ^7^. The report published on European HIV cohorts revealed similar trends of ADCs and NADCs in patients with HIV over the period of 2001 to 2012 ^8^. The report further emphasizes that the decline in ADCs is due to the use of cART, which effectively suppressed viral replication and reversed immunosuppression in patients with HIV. The increase in NADCs could be due to the higher life expectancy of PLWHAs receiving cART and to other risk factors for cancer such as high smoking rate and heavy alcohol drinking. A study of Spanish HIV-infected patients in 2017 reported hepatocellular carcinoma (20.5%), lung (18.7%), head and neck (11.9%), and anal (10.5%) as the most common types of NADCs ^9^. In the African HIV population, which comprises about 50% of global HIV patients, NADCs were observed to be increased by 33.8% from 2002 to 2014 ^10^. In this review, we will discuss the recent advances in cancers, primarily the extent of cancer incidence, prevalence, and progression among HIV patients as a consequence of smoking.

## Prevalence of smoking in HIV-infected patients

In a cross-sectional survey conducted on 419,945 patients with HIV in the US in 2015, 42.4% were current smokers, 20.3% were former smokers, and 37.3% were non-smokers ^11^. According to the study, the prevalence of smoking in patients with HIV (42.4%) was more than two times higher than the uninfected US adult population (20.6%). A similar trend of smoking prevalence was observed in a study surveying HIV-infected patients in New York in 2016, which found the association of smoking with poor health outcomes among PLWHAs ^12^. A study conducted in Ontario, Canada, in 2008 reported that 39.3% of the patients with HIV were smokers, which declined by 1.6% by 2014. However, the rate of smoking remained higher in the HIV population than in the general population ^13^. About 33% of patients with HIV in a South African study were also found to be smokers ^14^. Furthermore, the prevalence of smoking is much higher among male HIV patients who have sex with men (MSM) than it is among uninfected men ^15– 17^. Jin *et al*. performed a meta-analysis of 102 studies that included only Chinese PLWHAs, and the authors reported the prevalence of smoking to be 41.1% among the study population ^18^. Table 1 summarizes the prevalence of smoking among patients with HIV in different countries (most of the patients are from the US) from 2014 to 2017 ^19– 21^, showing that smoking is highly prevalent in HIV-infected populations.

**Table 1.** Prevalence of smoking among HIV-infected patients.

Authors	Date of publication	Location	Patients with HIV, number	HIV-infected smokers, percentage
Mdodo *et al.* ^11^	2015	US	419,945	42.4
Hile *et al*. ^12^	2016	US	14,713	40
Bekele *et al*. ^13^	2017	Canada	4,473	39.3
Akhtar-Khaleel *et al*. ^16^	2016	US	6,577	36.9
Pacek *et al*. ^19^	2014	US	358	75
Pollack *et al*. ^21^	2017	Vietnam	636	36.3
Murrison *et al*. ^14^	2016	South Africa	146	33
Brath *et al*. ^20^	2016	Austria and Germany	447	49.4

It can be noted that the literatures within the table have performed analysis independent of the information from other literatures within the table. Papers with reference number 11, 12, 13, 14, 16, 19, 20, 21, and 22 used original information. However, in the case of meta-analysis in some literatures (papers with reference 18 and 23), they have information from literatures present outside the table.

## Cancer risk in HIV-infected smokers

Several reports suggest that smoking is a risk factor for cancer development in PLWHAs ^18, 24^. A study by Helleberg *et al*. on 3,503 HIV-infected patients and 12,979 uninfected individuals in Denmark concluded that the risk of smoking-related cancers is much higher in HIV-infected smokers compared with both the general population and HIV-infected non-smokers. The study also found that about 23% of the cancers in the HIV-infected population were related to smoking ^24^. The authors classified the smoking-related cancers as lung cancer, head and neck cancer (HNC), esophagus cancer, and bladder cancer on the basis of the International Classifications of Diseases, 10th version (ICD-10). According to the study, the incidence rate ratios (IRRs) of smoking-related cancer associated with current smoking were 21.35 in patients with HIV and 4.12 within the general population. Similarly, another study performed by the same researchers on HIV-infected patients in Europe and North America reported that the mortality rate ratio for HIV-infected smokers versus non-smokers due to NADCs was 3.13, which also indicates smoking as an important risk factor for cancers in patients with HIV ^25^.

## Smoking-induced cancers in HIV-infected smokers

Shepherd *et al*. conducted a study in HIV cohorts across 33 European countries, which indicated the significant association of smoking with infection-unrelated malignancies (IURMs) (IRR = 1.75 when compared with never-smokers) rather than infection-related malignancies (IRMs) ^8^. The study classified virus-induced cancers caused by EBV (Hodgkin’s/non-Hodgkin’s lymphoma), hepatitis B and C viruses (liver cancer), HHV8 (Kaposi’s sarcoma), and HPV (cervical, anal, and stomach cancer) as IRMs. IURMs included lung, prostate, colorectal, and breast cancer. However, several reports in the literature suggest that smoking is also associated with some definitely virally induced cancers such as HNC and cervical and liver cancer and some potentially virally induced cancers such as esophageal cancer ^26– 31^. In Table 2, we have summarized the incidence rate (IR) of the smoking-associated cancers among HIV-infected patients in the last four years (2014–2017) ^6, 22, 24, 26, 28^. In general, the rates of incidence of lung cancer, anal cancer, HNC, cervical cancer, and esophageal cancer were 66–175, 76–146.8, 24–80, 21–506, and 12–21 per 100,000 person-years (PY), respectively. In the following sections, we will briefly discuss the recent findings on risk, prevalence, and progression of the most common cancers in HIV-positive smokers.

**Table 2.** Incidence of cancers in HIV-infected patients.

Authors	Date of publication	Location	Cancer type	Incidence rate, cancers per 100,000 person-years
Helleberg *et al*. ^24^	2014	Denmark	Lung	91
Hessol *et al*. ^26^	2015	US	Lung	119
Marcus *et al*. ^32^	2017	US	Lung	66
Park *et al*. ^23^	2016	US	Lung	175
Helleberg *et al*. ^24^	2014	Denmark	Esophageal	21
Park *et al*. ^23^	2016	US	Esophageal	12
Park *et al*. ^23^	2016	US	Non-HIV-related oral cavity and pharynx	24
Helleberg *et al*. ^24^	2014	Denmark	Head and neck	80
Chiao *et al*. ^22^	2014	US	Anal	134.3–146.8
Park *et al*. ^23^	2016	US	Anal	76
Ekanem and Parkin ^31^	2016	Nigeria	Cervix	21
Helleberg *et al*. ^24^	2014	Denmark	Cervix	25
Rohner *et al*. ^34^	2017	South Africa	Cervix	506

It can be noted that the literatures within the table have performed analysis independent of the information from other literatures within the table. Papers with reference number 24, 26, 31, 22, 32, and 34 used original information. However, the paper with reference 23 has performed meta-analysis based on information from literatures present outside the table.

## Lung cancer

The IRR for lung cancer is about 1.7–2 in HIV-infected smokers compared with the general population ^23, 32^. Hessol *et al*. reported higher incidence of lung cancer in HIV-infected smokers (IR/100,000 PY = 119) than in uninfected smokers (IR/100,000 PY = 45) ^26^. According to Makinson *et al*., HIV-infected smokers tend to suffer from lung cancer at a relatively younger age than the uninfected population ^33^. Reddy *et al*. used the Cost-Effectiveness of Preventing AIDS Complications (CEPAC)-US model to estimate the cumulative lung cancer mortality rate in 40-year-old men who were HIV-positive smokers. They estimated that the lung cancer mortality rate for current smokers in the study group was about 18 times higher than for individuals who had never smoked and about 3.8 times higher than those who quit smoking at the age of 40 years ^35^. However, the lung cancer burden in patients with HIV has declined (−2.8%) in the past few years in the US ^7^ and this could be due to the decrease in the smoking prevalence over the period. The analysis of lung histology in American patients with HIV revealed the most common types of lung cancer observed in American PLWHAs to be adenocarcinoma followed by squamous cell carcinoma and other non-small cell carcinoma ^26^. A similar study conducted on Chinese cohorts by Cheng *et al*. suggested that the majority (85%) of the HIV patients with lung cancer had non-small cell carcinoma and that nearly half of the study groups were at stage IIIb and IV of lung cancer ^36^.

The study by Marcus *et al*. in 2017 showed that the lung cancer rate ratio decreased from 1.4 to 1.2 when they calculated the lung cancer risk after adjustment for pneumonia ^32^. Furthermore, the study indicated HIV-mediated immunosuppression as a lung cancer risk factor in patients with HIV. Hence, factors other than smoking such as HIV-induced immunosuppression and history of lung diseases such as AIDS-related pneumonia also contribute as cumulative risk factors for lung cancer in PLWHAs.

## Head and neck cancer

HNC includes the cancers of larynx, oropharynx, oral cavity, and nasal cavity. The IR of HNC in HIV-infected smokers in the US ranges from 1.3 to 1.8 per 100,000 PY ^23^. A study by Faggons *et al*. on African patients with HIV suggested that 43% of the patients with HNC were tobacco smokers ^37^. The study also pointed out that cancers of the larynx, oropharynx, and oral cavity were the most common among HIV-positive smokers. Another study performed by D’Souza *et al*. among American HIV-HNC patients also reported about four times greater prevalence of smoking among HIV-positive patients with HNC compared with HIV-negative individuals with HNC ^38^. Furthermore, in the same study group, they observed that HIV patients suffered from HNC at a younger age (median age = 50 years) compared with the uninfected population (median age = 62 years). The most common type of HNCs observed was cancer of the oral cavity followed by oropharyngeal cancer and larynx cancer. Interestingly, the authors also observed a higher prevalence of HNCs in HIV patients who were currently on cART (77%) compared with those who were not (23%). Since HPV was detected in about 30% of the HIV patients with HNC (primarily in oropharyngeal cancers), the study implicated HPV infection, in addition to tobacco smoking, as a risk factor for HNC in patients with HIV.

## Esophageal cancer

The study conducted by McCormack *et al*. in six sub-Saharan African countries showed a high association of tobacco smoking with the risk of esophageal cancer (odds ratio [OR] = 2.6–8.0) ^39^. The study also confirmed that most of the patients in their study were HIV-positive. Another study in Denmark also suggested that PLWHAs are at higher risk of esophageal cancer (IR/100,000 PY = 8.0) compared with uninfected individuals ^24^. Several studies have reported the association of smoking with esophageal cancer risk in HIV-infected patients ^29, 40^. Moses *et al*. reported an OR of about 2.02 for smokers versus non-smokers for esophageal cancer in HIV-infected patients in Malawi ^40^. Another study conducted in Zambia by Kayamba *et al*. also reported that cigarette smoking, independent of HIV infection, conferred high risk of esophageal cancer in HIV-infected patients (OR = 3.5) ^29^. Furthermore, the study reported a higher prevalence of esophageal squamous cell carcinoma at the middle section (60.4%) of the esophagus, followed by the distal (26.4%) and proximal (10.6%) sections ^41^.

## Anal cancer

Most studies published in recent years report that HPV is a major risk factor for the prevalence of anal cancer in patients with HIV, especially among MSM ^42– 44^. Some studies also indicate smoking as an indirect risk for anal cancer, as it triggers HPV replication ^45, 46^. A study performed in HIV-infected women by Gaisa *et al*. reported that the risk of anal cancer doubled in HIV-infected smokers ^47^. Another study from Wieland *et al*. suggested that smoking is a risk factor for anal cancer in HIV-infected men, especially MSM ^45^. Higher HPV DNA loads, higher prevalence of high/low-grade intraepithelial lesions, and greater incidence of anal dysplasia were observed in HIV-infected MSM who were smokers compared with those who were non-smokers. Although the anal cancer rate has risen over the last decade, the cancer survival rate has also increased by 62–67% after chemoradiotherapy. However, the recurrence rates for anal cancer are still high, ranging from 30% to 60% in HIV patients compared with uninfected populations, and this is probably due to lower tolerance of chemoradiotherapy ^48^.

## Cervical cancer

Cervical cancer is an ADC with higher risk and prevalence among HIV-1-infected women compared with uninfected women ^49^. The IR of cervical cancer is about three times higher in HIV-infected women than in HIV-uninfected women ^50^. Although HPV infection is the major cause of cervical cancer, several studies in the past have implicated cigarette smoking as a cofactor for cervical cancer in both HIV-infected and uninfected women ^51, 52^. Moreover, most of the recent studies have emphasized HPV infection, immunosuppression by HIV, age, abortion, and vaginal abnormalities as major risk factors for cervical cancer in HIV-infected women ^50, 53– 55^. Only a few recent reports associate smoking with cervical cancer risk in HIV-infected women. A study of HIV-infected sub-Saharan African women by Rohner *et al*. reported that the IR of cervical cancer in HIV-infected women is 506/100,000 PY, which is higher than in European populations ^34^.

## Conclusions

Smoking is highly prevalent among HIV-infected patients compared with the general population. Therefore, HIV-infected individuals are at higher risk of tobacco-related health complications, particularly cancers. Several studies have shown a strong association of smoking with cancer incidence in patients with HIV. The recent studies have reported the predominant prevalence of lung, head and neck, esophageal, anal, and cervical cancers in HIV-infected smokers. Smoking cessation intervention could be a crucial preventive measure to reduce the incidence of cancers in PLWHAs. In the cases of HIV-infected patients already suffering from cancer, smoking cessation may help to decelerate the progression or reduce the severity of the disease. Awareness, counseling, and campaigns regarding the hazards of smoking are crucial to motivate and assist HIV-infected smokers to quit smoking. In addition to motivational efforts, medical treatments to alleviate tobacco addiction, such as the use of nicotine alternatives like nicotine patches, gums, inhalers, lozenges, and nasal sprays, should be encouraged. Alternatively, medications such as bupropion and varenicline may also help reduce the incidence of cigarette smoking. Finally, cancer prevalence and severity are more prominent among untreated PLWHAs rather than those receiving cART. Therefore, early initiation of cART and early diagnostic screenings for cancer in PLWHAs are important strategies to reduce cancer risks in these patients.

## References

[1] Robbins HA Pfeiffer RM Shiels MS: Excess cancers among HIV-infected people in the United States. *J Natl Cancer Inst.* 2015;107(4): pii: dju503. 10.1093/jnci/dju503 25663691 PMC4334816

[2] Vandenhende MA Roussillon C Henard S: Cancer-Related Causes of Death among HIV-Infected Patients in France in 2010: Evolution since 2000. *PLoS One.* 2015;10(6):e0129550. 10.1371/journal.pone.0129550 26083524 PMC4470800

[3] Kahn JA Rudy BJ Xu J: Prevalence and risk factors for oral DNA tumor viruses in HIV-infected youth. *J Med Virol.* 2016;88(11):1944–52. 10.1002/jmv.24555 27096166 PMC5008985

[4] Pierangeli A Antonelli G Gentile G: Immunodeficiency-associated viral oncogenesis. *Clin Microbiol Infect.* 2015;21(11):975–83. 10.1016/j.cmi.2015.07.009 26197213

[5] Rubinstein PG Aboulafia DM Zloza A: Malignancies in HIV/AIDS: from epidemiology to therapeutic challenges. *AIDS.* 2014;28(4):453–65. 10.1097/QAD.0000000000000071 24401642 PMC4501859

[6] Chiu CG Smith D Salters KA: Overview of cancer incidence and mortality among people living with HIV/AIDS in British Columbia, Canada: Implications for HAART use and NADM development. *BMC Cancer.* 2017;17(1):270. 10.1186/s12885-017-3229-1 28410587 PMC5391557

[7] Robbins HA Shiels MS Pfeiffer RM: Epidemiologic contributions to recent cancer trends among HIV-infected people in the United States. *AIDS.* 2014;28(6):881–90. 10.1097/QAD.0000000000000163 24300545 PMC5015650

[8] Shepherd L Borges Á Ledergerber B: Infection-related and -unrelated malignancies, HIV and the aging population. *HIV Med.* 2016;17(8):590–600. 10.1111/hiv.12359 26890156

[9] Rodríguez Arrondo F von Wichmann MÁ Camino X: A case-control study of non-AIDS-defining cancers in a prospective cohort of HIV-infected patients. *Med Clin (Barc).* 2018;150(8):291–6. 10.1016/j.medcli.2017.03.032 28528797

[10] Campbell JA Soliman AS Kahesa C: Changing Patterns of lung, liver, and head and neck non-AIDS-defining cancers relative to HIV status in Tanzania between 2002-2014. *Infect Agent Cancer.* 2016;11:58. 10.1186/s13027-016-0106-5 27895703 PMC5117569

[11] Mdodo R Frazier EL Dube SR: Cigarette smoking prevalence among adults with HIV compared with the general adult population in the United States: cross-sectional surveys. *Ann Intern Med.* 2015;162(5):335–44. 10.7326/M14-0954 25732274

[12] Hile SJ Feldman MB Alexy ER: Recent Tobacco Smoking is Associated with Poor HIV Medical Outcomes Among HIV-Infected Individuals in New York. *AIDS Behav.* 2016;20(8):1722–9. 10.1007/s10461-015-1273-x 26837623 PMC4942487

[13] Bekele T Rueda S Gardner S: Trends and Correlates of Cigarette Smoking and Its Impacts on Health-Related Quality of Life Among People Living with HIV: Findings from the Ontario HIV Treatment Network Cohort Study, 2008-2014. *AIDS Patient Care STDS.* 2017;31(2):49–59. 10.1089/apc.2016.0174 28170303

[14] Bronner Murrison L Martinson N Moloney RM: Tobacco Smoking and Tuberculosis among Men Living with HIV in Johannesburg, South Africa: A Case-Control Study. *PLoS One.* 2016;11(11):e0167133. 10.1371/journal.pone.0167133 27893799 PMC5125673

[15] Akhtar-Khaleel WZ Cook RL Shoptaw S: Long-Term Cigarette Smoking Trajectories Among HIV-Seropositive and Seronegative MSM in the Multicenter AIDS Cohort Study. *AIDS Behav.* 2016;20(8):1713–21. 10.1007/s10461-016-1343-8 26922718 PMC4945456

[16] Akhtar-Khaleel WZ Cook RL Shoptaw S: Trends and Predictors of Cigarette Smoking Among HIV Seropositive and Seronegative Men: The Multicenter Aids Cohort Study. *AIDS Behav.* 2016;20(3):622–32. 10.1007/s10461-015-1099-6 26093780 PMC4760908

[17] Hirshfield S Schrimshaw EW Stall RD: Drug Use, Sexual Risk, and Syndemic Production Among Men Who Have Sex With Men Who Engage in Group Sexual Encounters. *Am J Public Health.* 2015;105(9):1849–58. 10.2105/AJPH.2014.302346 25713951 PMC4539820

[18] Jin ZY Liu X Ding YY: Cancer risk factors among people living with HIV/AIDS in China: a systematic review and meta-analysis. *Sci Rep.* 2017;7(1): 4890. 10.1038/s41598-017-05138-x 28687813 PMC5501798

[19] Pacek LR Latkin C Crum RM: Current cigarette smoking among HIV-positive current and former drug users: associations with individual and social characteristics. *AIDS Behav.* 2014;18(7):1368–77. 10.1007/s10461-013-0663-1 24287787 PMC4037399

[20] Brath H Grabovac I Schalk H: Prevalence and Correlates of Smoking and Readiness to Quit Smoking in People Living with HIV in Austria and Germany. *PLoS One.* 2016;11(2):e0150553. 10.1371/journal.pone.0150553 26919722 PMC4771118

[21] Pollack TM Duong HT Pham TT: Cigarette smoking is associated with high HIV viral load among adults presenting for antiretroviral therapy in Vietnam. *PLoS One.* 2017;12(3):e0173534. 10.1371/journal.pone.0173534 28267790 PMC5340371

[22] Chiao EY Hartman CM El-Serag HB: The impact of HIV viral control on the incidence of HIV-associated anal cancer. *J Acquir Immune Defic Syndr.* 2013;63(5):631–8. 10.1097/QAI.0b013e3182968fa7 23614995 PMC3797186

[23] Park LS Tate JP Sigel K: Time trends in cancer incidence in persons living with HIV/AIDS in the antiretroviral therapy era: 1997-2012. *AIDS.* 2016;30(11):1795–806. 10.1097/QAD.0000000000001112 27064994 PMC4925286

[24] Helleberg M Gerstoft J Afzal S: Risk of cancer among HIV-infected individuals compared to the background population: impact of smoking and HIV. *AIDS.* 2014;28(10):1499–508. 10.1097/QAD.0000000000000283 24785952

[25] Helleberg M May MT Ingle SM: Smoking and life expectancy among HIV-infected individuals on antiretroviral therapy in Europe and North America. *AIDS.* 2015;29(2):221–9. 10.1097/QAD.0000000000000540 25426809 PMC4284008

[26] Hessol NA Martínez-Maza O Levine AM: Lung cancer incidence and survival among HIV-infected and uninfected women and men. *AIDS.* 2015;29(10):1183–93. 10.1097/QAD.0000000000000690 25888645 PMC4457511

[27] Beachler DC D'Souza G: Oral human papillomavirus infection and head and neck cancers in HIV-infected individuals. *Curr Opin Oncol.* 2013;25(5):503–10. 10.1097/CCO.0b013e32836242b4 23852381 PMC3896303

[28] Kolly P Knöpfli M Dufour JF: Effect of smoking on survival of patients with hepatocellular carcinoma. *Liver Int.* 2017;37(11):1682–7. 10.1111/liv.13466 28467657

[29] Kayamba V Bateman AC Asombang AW: HIV infection and domestic smoke exposure, but not human papillomavirus, are risk factors for esophageal squamous cell carcinoma in Zambia: a case-control study. *Cancer Med.* 2015;4(4):588–95. 10.1002/cam4.434 25641622 PMC4402073

[30] Fletcher FE Vidrine DJ Tami-Maury I: Cervical cancer screening adherence among HIV-positive female smokers from a comprehensive HIV clinic. *AIDS Behav.* 2014;18(3):544–54. 10.1007/s10461-013-0480-6 23605155 PMC4383031

[31] Ekanem IO Parkin DM: Five year cancer incidence in Calabar, Nigeria (2009-2013). *Cancer Epidemiol.* 2016;42:167–72. 10.1016/j.canep.2016.04.014 27164305

[32] Marcus JL Leyden WA Chao CR: Immunodeficiency, AIDS-related pneumonia, and risk of lung cancer among HIV-infected individuals. *AIDS.* 2017;31(7):989–93. 10.1097/QAD.0000000000001434 28252529 PMC5373807

[33] Makinson A Eymard-Duvernay S Raffi F: Feasibility and efficacy of early lung cancer diagnosis with chest computed tomography in HIV-infected smokers. *AIDS.* 2016;30(4):573–82. 10.1097/QAD.0000000000000943 26829006

[34] Rohner E Sengayi M Goeieman B: Cervical cancer risk and impact of Pap-based screening in HIV-positive women on antiretroviral therapy in Johannesburg, South Africa. *Int J Cancer.* 2017;141(3):488–96. 10.1002/ijc.30749 28440019 PMC5504282

[35] Reddy KP Kong CY Hyle EP: Lung Cancer Mortality Associated With Smoking and Smoking Cessation Among People Living With HIV in the United States. *JAMA Intern Med.* 2017;177(11):1613–21. 10.1001/jamainternmed.2017.4349 28975270 PMC5675744

[36] Cheng Z Shan F Liu J: Clinical and computed tomography findings in Chinese lung cancer patients with HIV infection: A multi-center study. *Thorac Cancer.* 2017;8(3):238–45. 10.1111/1759-7714.12429 28294549 PMC5415480

[37] Faggons CE Mabedi C Shores CG: Review: Head and neck squamous cell carcinoma in sub-Saharan Africa. *Malawi Med J.* 2015;27(3):79–87. 10.4314/mmj.v27i3.2 26715951 PMC4688867

[38] D'Souza G Carey TE William WN Jr: Epidemiology of head and neck squamous cell cancer among HIV-infected patients. *J Acquir Immune Defic Syndr.* 2014;65(5):603–10. 10.1097/QAI.0000000000000083 24326607 PMC3999230

[39] McCormack VA Menya D Munishi MO: Informing etiologic research priorities for squamous cell esophageal cancer in Africa: A review of setting-specific exposures to known and putative risk factors. *Int J Cancer.* 2017;140(2):259–71. 10.1002/ijc.30292 27466161 PMC5763498

[40] Moses A Mwafongo A Chikasema M: Risk factors for common cancers among patients at Kamuzu Central Hospital in Lilongwe, Malawi: A retrospective cohort study. *Malawi Med J.* 2017;29(2):136–41. 28955421 10.4314/mmj.v29i2.11 PMC5610284

[41] Loots E Sartorius B Madiba TE: Oesophageal squamous cell cancer in a South African tertiary hospital: a risk factor and presentation analysis. *S Afr J Surg.* 2017;55(3):42–6. 28876564

[42] Cranston RD Althouse AD van Griensven F: Prevalence of Anal Human Papillomavirus Vaccine Types in the Bangkok Men Who Have Sex With Men Cohort Study. *Sex Transm Dis.* 2015;42(12):671–6. 10.1097/OLQ.0000000000000372 26562695

[43] Hu Y Qian H Sun J: Anal human papillomavirus infection among HIV-infected and uninfected men who have sex with men in Beijing, China. *J Acquir Immune Defic Syndr.* 2013;64(1):103–14. 10.1097/QAI.0b013e31829b6298 23732908 PMC3780393

[44] Nagata N Watanabe K Nishijima T: Prevalence of Anal Human Papillomavirus Infection and Risk Factors among HIV-positive Patients in Tokyo, Japan. *PLoS One.* 2015;10(9):e0137434. 10.1371/journal.pone.0137434 26368294 PMC4569050

[45] Wieland U Hellmich M Wetendorf J: Smoking and anal high-risk human papillomavirus DNA loads in HIV-positive men who have sex with men. *Int J Med Microbiol.* 2015;305(7):689–96. 10.1016/j.ijmm.2015.08.019 26319939

[46] Schabath MB Villa LL Lin H: A prospective analysis of smoking and human papillomavirus infection among men in the HPV in Men Study. *Int J Cancer.* 2014;134(10):2448–57. 10.1002/ijc.28567 24222514 PMC3949156

[47] Gaisa M Ita-Nagy F Sigel K: High Rates of Anal High-Grade Squamous Intraepithelial Lesions in HIV-Infected Women Who Do Not Meet Screening Guidelines. *Clin Infect Dis.* 2017;64(3):289–94. 10.1093/cid/ciw729 27965301 PMC6366052

[48] Spano JP Poizot-Martin I Costagliola D: Non-AIDS-related malignancies: expert consensus review and practical applications from the multidisciplinary CANCERVIH Working Group. *Ann Oncol.* 2016;27(3):397–408. 10.1093/annonc/mdv606 26681686

[49] Ghebre RG Grover S Xu MJ: Cervical cancer control in HIV-infected women: Past, present and future. *Gynecol Oncol Rep.* 2017;21:101–8. 10.1016/j.gore.2017.07.009 28819634 PMC5548335

[50] Abraham AG D'Souza G Jing Y: Invasive cervical cancer risk among HIV-infected women: a North American multicohort collaboration prospective study. *J Acquir Immune Defic Syndr.* 2013;62(4):405–13. 10.1097/QAI.0b013e31828177d7 23254153 PMC3633634

[51] Lea JS Coleman R Kurien A: Aberrant *p16* methylation is a biomarker for tobacco exposure in cervical squamous cell carcinogenesis. *Am J Obstet Gynecol.* 2004;190(3):674–9. 10.1016/j.ajog.2003.09.036 15041998

[52] Castellsagué X Muñoz N: Chapter 3: Cofactors in human papillomavirus carcinogenesis--role of parity, oral contraceptives, and tobacco smoking. *J Natl Cancer Inst Monographs.* 2003;2003(31):20–8. 10.1093/oxfordjournals.jncimonographs.a003477 12807941

[53] Clifford GM Tully S Franceschi S: Carcinogenicity of Human Papillomavirus (HPV) Types in HIV-Positive Women: A Meta-Analysis From HPV Infection to Cervical Cancer. *Clin Infect Dis.* 2017;64(9):1228–35. 10.1093/cid/cix135 28199532 PMC5399941

[54] Ononogbu U Almujtaba M Modibbo F: Cervical cancer risk factors among HIV-infected Nigerian women. *BMC Public Health.* 2013;13:582. 10.1186/1471-2458-13-582 23767681 PMC3728111

[55] Liu E McCree R Mtisi E: Prevalence and risk factors of cervical squamous intraepithelial lesions among HIV-infected women in Dar es Salaam, Tanzania. *Int J STD AIDS.* 2016;27(3):219–25. 10.1177/0956462415584466 25957324 PMC11340030

